# From paternalism to partnership: A patient-centred view of shared decision-making in primary health care

**DOI:** 10.4102/phcfm.v18i1.5394

**Published:** 2026-09-11

**Authors:** Shavania Ragavan, Michéle Torlutter, Deidré Pretorius

**Affiliations:** 1 Department of Family Medicine and Primary Care, Faculty of Health Sciences, University of the Witwatersrand, Johannesburg, South Africa

**Keywords:** shared decision-making, primary health care, barriers, facilitators, communication

## Abstract

**Background:**

Shared decision-making (SDM) has been advocated in the past decade for patient-centredness and autonomy. Research in primary health care (PHC) within South Africa lacks information about the uptake of SDM.

**Aim:**

This study aimed to determine whether SDM occurred in PHC and the barriers, facilitators and sociodemographic factors that affect its uptake in the South African PHC setting.

**Setting:**

The Alexandra Community Healthcare Centre (CHC) and Chiawelo CHC are facilities that serve the communities of Alexandra and Chiawelo, respectively.

**Methods:**

The study was an analytical cross-sectional survey with a sample size of 391 participants conducted at two CHCs in the Johannesburg Metro district. A modified SDM Q-9 tool was used to determine if SDM occurred. This was followed by two more questionnaires that assessed patients’ preferred decision-making method and their perception of the barriers and facilitators of SDM. Analytical statistics, Pearson Chi^2^ testing and univariate and multivariate regression were used for data analysis.

**Results:**

Eighty-five per cent of respondents reported that SDM did not occur during their last consultation with a healthcare worker. The two most preferred consultation models were the SDM model (37%) and the paternalistic model (36%). Participants who completed secondary education (odds ratio [OR] = 2.82, 95% confidence interval [CI]: 1.10–7.23, *p*-value: 0.031) and tertiary education (OR = 6.28, 95% CI: 1.83–21.61, *p*-value: 0.004) were more likely to engage in SDM.

**Conclusion:**

More than a third of patients wanted to be involved in SDM, but it was not routinely practised during consultations. Patients with higher levels of education reported greater SDM. Healthcare workers must be more receptive and invite open dialogue to encourage SDM.

**Contribution:**

The information from this article can be used to strengthen undergraduate and postgraduate training around SDM, improve patient satisfaction and uptake of care.

## Introduction

Every human being of adult years and sound mind has a right to determine what shall be done with his own body.^1^

### Background

Shared decision-making (SDM) has become the preferred model for patient care as it supports patients’ fundamental rights of autonomy and bodily integrity by actively involving patients in their health decisions.^2^ Shared decision-making can be described as a collaborative process where clinicians and patients jointly make healthcare decisions, incorporating the best available evidence with the patient’s values, preferences and circumstances.^3^ Shared decision-making thus assists the clinician to appreciate the subjective aspect of the patient’s illness.^4^ The United Kingdom (UK) and United States (US) have seen progress in the implementation of SDM, which can be attributed to strong policy mandates, legal precedents, development of support tools and patient autonomy.^5^ Despite the benefits, there remains a massive gap between aspiration and practical application of SDM, specifically in developing countries such as South Africa.^6^ The four styles of decision-making that have been widely adopted in clinical care are the paternalistic model, the SDM model, the consumerist model and the interpretive model.^7^ The SDM model was developed in 1997.^8^ The model is comprised of four key elements, which include a clinician and a patient, both parties sharing information, and both parties trying to reach a consensus regarding the treatment plan.^9^ Shared decision-making honours a ‘patient-led consultation’ and ‘choice’, thus placing the patient at the core of patient-centred care.^10,11^ The informative model or consumerist model allows the patient to make the final decision about their healthcare based on all the information and choices presented to them by the healthcare worker (HCW). The paternalistic model allows the HCW to act as the patient’s ‘guardian’ and make decisions on the patient’s behalf without patient interaction. The interpretive model involves the patient presenting many different options to the HCW, and the HCW then advising them which option is the best based on the patient’s clinical condition and their expertise.^12^ The primary health care (PHC) setting is considered the starting point of SDM in a patient’s healthcare journey.^13^ Shared decision-making has been shown to increase patient satisfaction, which in turn improves treatment adherence and overall quality of life.^13,14^ Shared decision-making is also linked to decreased healthcare costs, prevention of unnecessary referrals to specialist care and promotion of patient autonomy.^15,16^ This reinforces the critical role of SDM in resource-limited healthcare facilities across South Africa.^4,17^ A study conducted in South Africa revealed that involving patients in their treatment plan increased their cooperation and understanding, thus highlighting SDM as a crucial component in the South African context.^18^ In Europe, a large population-based study revealed that most respondents (96%) preferred to be offered choices and be asked for their opinions.^19^ This contrasted with a study in West Africa, which found that the paternalistic model was still the preferred model and that not enough was being done in healthcare to advocate for the uptake of SDM or to promote healthcare training surrounding the SDM approach.^20^ A South African study found that almost half of the patients interviewed at a district hospital (46%) wanted to be involved in SDM. Despite these findings, doctors assumed that patients did not want to engage in this type of collaborative consultation due to lower educational and socioeconomic status.^21^ South African doctors do not readily provide information to patients who do not show interest in their treatment plans and assume that all patients want a paternalistic approach in their treatment decisions.^18^ A predominant factor influencing the uptake of SDM is gender, with women taking a more active role than men.^22^ Age was also found to be a significant influence, with younger patients assuming an active role.^22,23^ Furthermore, people with higher education wanted to be more involved in their treatment choices.^17^ The nature of the illness also determines the wish to engage in SDM, that is, the more life-threatening the illness, the less inclined a patient is to participate in SDM.^24^ Asian and African patients preferred family involvement in treatment decision-making.^23,25,26^ Family engagement in South African healthcare is often observed in primary health care, primarily because African cultural beliefs are rooted in the notion that a person’s identity is deeply embedded in their collective existence.^26^ A qualitative study conducted in London found that the major barriers to patients becoming involved in SDM included difficulties in communication, noisy environments and difficulty involving a carer or escort.^27^ Healthcare practitioners also cite time constraints as reasons for not engaging with patients and not providing concise patient education regarding treatment and procedures.^18^ The main facilitators that prompted the uptake of SDM included early involvement in decision-making, involvement of the whole clinical team, positive relationships with staff members, good administration and a friendly doctor.^27^ In another subset of patients, including patients with lower literacy levels, it has been found that the use of decision aids could assist with language and cultural barriers.^8,18^

Africa is lagging in the clinical application of the SDM model.^15^ Thus far, there has been no research conducted in South Africa that focused on the implementation of SDM in primary health care or the barriers and facilitators that affect its uptake. In the African healthcare system, a massive socioeconomic and literacy divide exists, which poses a major challenge to implementing SDM. In an ideal situation, SDM would form a compulsory component of a consultation, as it forms the basis of active participation, which reinforces the patient’s importance in their treatment decision.^28^ Shared decision-making significantly improves medication adherence among individuals with chronic conditions, including diabetes and hypertension, human immunodeficiency virus (HIV), and even patients undergoing treatment for cancer.^29^ This research aimed to determine the proportion of patients reporting SDM and highlight the barriers, facilitators and sociodemographic determinants of patient engagement in SDM.

## Research methods and design

### Study design

The study was an analytical cross-sectional survey conducted at two community healthcare clinics in the Johannesburg Metropolitan District, which explored the patient perspective.

### Setting

The Alexandra Community Healthcare Centre (CHC) and Chiawelo Community Healthcare Clinic are public healthcare facilities that serve the communities of Alexandra and Chiawelo, respectively. Hereafter, the clinics will be referred to by number to ensure anonymity of the individuals working there. The study was conducted in the chronic care sections of both clinics. The non-communicable diseases seen at these clinics are comparable to the national non-communicable disease profile.^30^ These facilities offer an array of services, including acute care, chronic care, child and maternity care, HIV management, prevention services, radiology and rehabilitation services. The doctor–patient ratio, as per Gauteng provincial averages, was estimated at 1:4024. According to the 2017 General Household Survey, 71.2% of the population use public providers as a first choice of consultation when ill, with PHC serving as the first point of entry into the health system.^31^

### Study population

The total number of patients registered at Site 1 in the general chronic outpatient department was 12 000, and the number of registered patients at Site 2 in the general chronic outpatient department was 33 585 annually. Inclusion criteria were all patients older than 18 years of age who consulted a HCW in the morning. Patients who had poorly controlled psychiatric conditions and patients who needed acute emergency assistance were excluded from the study.

### Sample size

The sample size was calculated using a two-step sample calculation.^32^ The first formula was used to calculate the total sample size with a *p*-value of < 0.05, a confidence interval (CI) of 95% and a 0.05 margin of error. The required sample size was calculated at 384. At the end of the study, our sample size was 391, resulting from 103 participants at Site 1 and 288 at Site 2.

### Sampling

Non-probability consecutive sampling was utilised for this study.^12^ After concluding their consultation with a HCW, all participants who fell within the inclusion criteria were approached in order of their position in the pharmacy queue to be part of the study.

### Data collection tools

The English version of the 9-item Shared Decision-Making Questionnaire (SDM Q-9) is a validated tool used to assess whether SDM was done during the last consultation.^33^ The tool has an excellent internal consistency (Cronbach’s alpha > 0.9) and construct validity. The tool has a 6-point Likert scale with a final score of 45 points; a higher score implies that SDM was done.^33^ It comes in a doctor’s and a patient’s version and has been translated into many different languages.

The pilot study conducted on a smaller sample in a different CHC revealed that South African patients found the original 6-point Likert scale challenging to interpret. In our study, we had opted to use the SDM Q-9 with 4 options on the Likert scale (strongly disagree, disagree, agree, strongly agree) rather than the 6-option SDM Q-9 (completely disagree, totally disagree, somewhat disagree, somewhat agree, totally agree, completely agree). The 4-option SDM Q-9 scored a Cronbach’s alpha of 0.92, which shows excellent internal consistency, validity and reliability.^33,34^ Each option on the Likert scale was scored 1, 2, 3 or 4, giving a total score at the end of each questionnaire (36 points).^34^ We then followed Rodenburg-Vandenbussche et al.^35^ and categorised all scores falling below the midpoint (< 50%) as ‘no’, meaning no SDM took place. All scores that fell on or above the midpoint (≥ 50%) were labelled as ‘yes’, meaning that SDM took place. The preferred decision-making style questionnaire required the patient to express which was their preferred decision-making model. Patients were shown pictures depicting a scenario of each decision-making model. The options presented to patients included the SDM model, paternalistic model, informative model and interpretive model. The final questionnaire looked at the commonly reported barriers and facilitators to SDM in South African PHC settings and asked participants to choose three barriers and three facilitators from pictures based on their experience. They were then asked to put these pictures in order of the influence they had on SDM in their most recent consultation. (worst to least bad and best to least good, respectively). Patients were provided with options so that data could be statistically analysed in a consistent manner.

### Data collection

Patients were sampled between February 2024 and April 2024 every Monday, Wednesday and Thursday between 9:00 and 11:00. Patients meeting the inclusion criteria were recruited from the pharmacy waiting area. Patients attending the outpatient department were asked if they would like to participate in the study and were assured that their place in the pharmacy queue would be kept. They were approached in a consecutive manner based on their position in the pharmacy queue. This ensured that everyone had an equal opportunity to be included in the study.^36^ Once they agreed, they were taken to a private area in the clinic so that confidentiality and privacy could be maintained. Potential participants were requested to read the information sheet before signing the electronic informed consent form. If they consented, they proceeded to complete the questionnaires on a tablet linked to the electronic questionnaires on the RedCap online survey platform.^37^ The researcher facilitated the process by managing the visual aids to ensure that all patients could understand the concepts referred to in the same manner. The researcher received assistance from two non-clinical staff members from each respective site to translate sentences into IsiZulu, the most widely spoken language in Gauteng.^38^ The assistants were asked to use a neutral tone and follow the questionnaires as they appeared on the online system so as not to influence the participants’ options. The researcher had to assist those who had trouble navigating the RedCap online system (submitting questionnaires or moving on to the next form). The patients were thanked and taken back to their place in the pharmacy queue (Figure 1).

**FIGURE 1:**
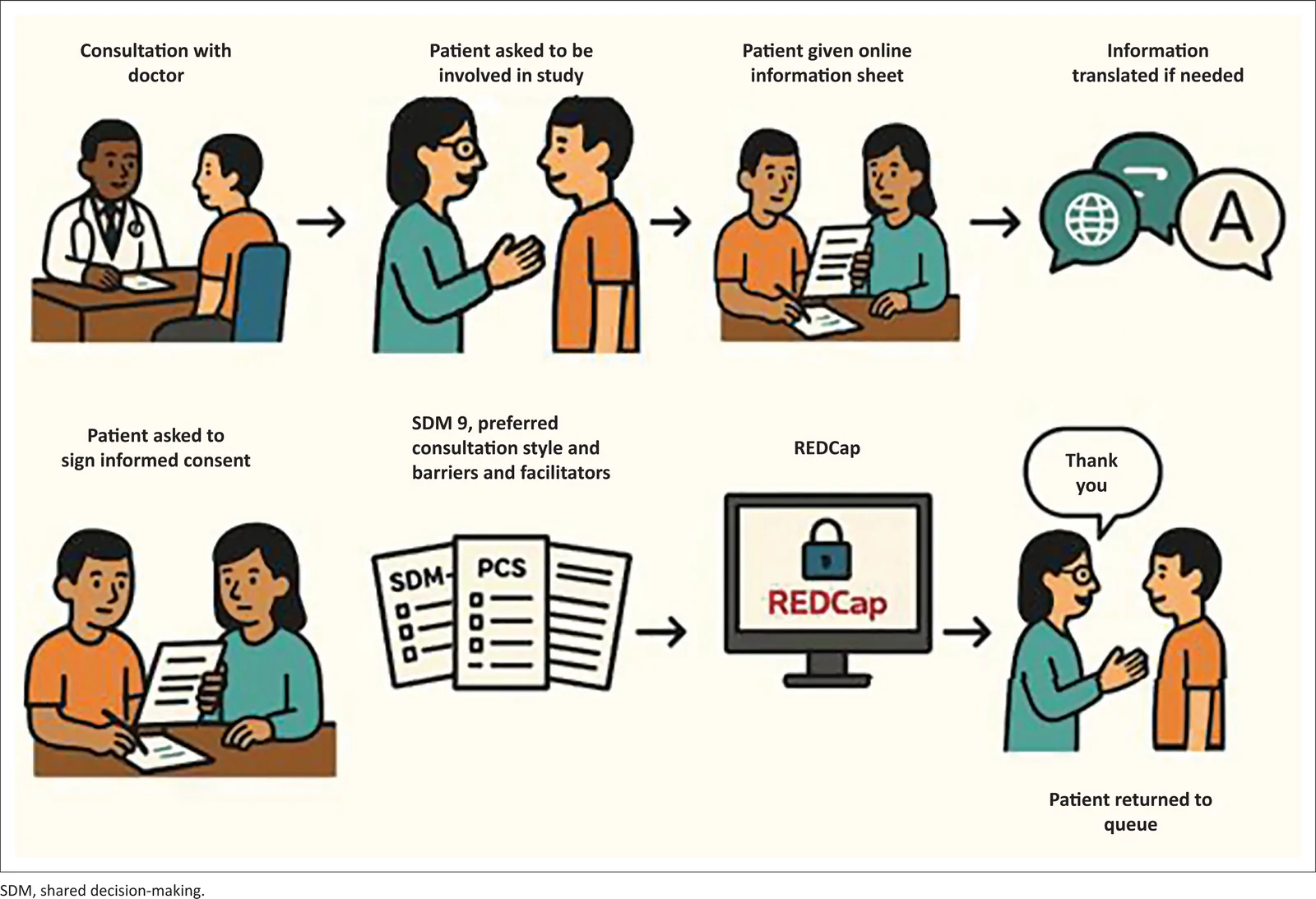
Data collection flow diagram.

### Data analysis

Data captured on RedCap were exported to Microsoft Excel before being uploaded to the STATA version 18 application for analysis.^39^ Data distribution was determined using skewness and scatter plots, as data were not normally distributed. Descriptive statistics were used for categorical variables, and the median and quartile ranges for continuous variables. The Pearson Chi^2^ test was utilised to determine the differences and statistical correlations between groups. Univariate logistic regression was used to determine the crude odds ratio (COR). The multivariate analysis provided us with the adjusted odds ratio (AOR). Variables with a *p* < 0.05 in the multivariate logistic regression were classified as statistically significant.

### Data management

The tablet used to collect data was password-protected, and data were backed up online on the Cloud. Every completed questionnaire was saved in the database, and a new, clean questionnaire was opened for the next patient. No identifiers were used to ensure that the study complied with the *Protection of Personal Information Act* (POPI) (Act No. 4 of 2013).^40^

### Ethical considerations

Ethical clearance to conduct this study was obtained from the University of the Witwatersrand, Human Research Ethics Committee (Medical) (No. M220311).

## Results

The overall sample size of the study was 391 participants. Two hundred and twenty-three (57%) participants were women. The calculated interquartile range (IQR) for age was 34–54 years, with the median being 43 years of age. The sample population had almost an equal number in each age quartile. Further age categorisation as per gender revealed that the median age in women was 42 years of age (IQR: 32–52) and, in men, it was 46 years of age (IQR: 36–55). Most of the participants completed secondary education (*n* = 262, 67%). When asked about employment, 43% (*n* = 167) were employed. More than half of the respondents were single (*n* = 217, 55%). Participants who were unmarried or cohabiting with their partner were included in this subcategory (Table 1).

**TABLE 1:** Demographic characteristics.

Characteristics	Level	*n*	%	Median	Q1–Q3
Gender	Men	168	43	-	-
	Women	223	57	-	-
Age per gender (years)	Men	-	-	46	36–55
	Women	-	-	42	32–52
Age (years)	< 34	100	26	-	-
	35–43	97	25	-	-
	44–53	100	26	-	-
	> 54	94	24	-	-
Home language	IsiXhosa	40	10	-	-
	IsiZulu	106	27	-	-
	Sesotho	51	13	-	-
	Setswana	36	9	-	-
	Tshivenda	39	10	-	-
	Tsonga	69	18	-	-
	Other (English, Sepedi, Chewa, IsiSwati, Ndebele, Shona, Swahili)	50	13	-	-
Highest level of education (completed)	Primary	90	23	-	-
	Secondary	262	67	-	-
	Tertiary	39	10	-	-
Health facility	Site 1	103	26	-	-
	Site 2	288	74	-	-
Employment	Employed	167	43	-	-
	Unemployed	136	35	-	-
	Grant	9	2	-	-
	Pension	33	8	-	-
	Self-employed	46	12	-	-
Marital status	Single	217	55	-	-
	Married	92	24	-	-
	Divorced	13	3	-	-
	Widowed	27	7	-	-
	Separated	42	11	-	-

The SDM (*n* = 146, 37%) and the paternalistic (*n* = 142, 36%) styles ranked the highest among the decision-making styles. Further categorising these preferences depicted that participants at Site 1 were more inclined to choose SDM (*n* = 64, 61%) as compared to Site 2, where the paternalistic approach was the highest ranked as compared to the other decision-making styles (*n* = 141, 49%). The results of the 9-item SDM questionnaire revealed that most patients did not experience SDM during their last consultation with a HCW (*n* = 331, 85%). These patient preferences and perspectives regarding SDM are illustrated in Table 2.

**TABLE 2:** Patient preferences and perspectives regarding decision-making.

Measured variable	Category	Frequency	
		*n*	%
Preferred consultation style combined	Shared decision-making	146	37
	Paternalistic	142	36
	Informative model	46	12
	Interpretive model	57	15
Highest ranking of preferred consultation style per site	Site 1	Shared decision-making:	
		64	62
	Site 2	Paternalistic consult:	
		141	49
Shared decision-making	No	331	85
	Yes	60	15

There was no significant association between experiencing SDM and gender (*p* = 0.95), age (*p* = 0.54), language (*p* = 0.26), study sites (*p* = 0.53), employment (*p* = 0.90) or marital status (*p* = 0.41). A significant association was identified between SDM and the overall level of education (*p* = 0.021). Respondents with tertiary education were most likely to report SDM (*n* = 10, 26%), compared to those who completed secondary (*n* = 43, 16%) or primary education (*n* = 7, 8%) (see Table 3).

**TABLE 3:** Demographic factors associated with shared decision-making.

Characteristic	Category	No		Yes		*p*-value
		*n*	%	*n*	%	
Gender	-	-	-	-	-	0.95
	Male	142	85	26	15	-
	Female	188	84	35	16	-
Age (in years)	-	-	-	-	-	0.54
	≤ 34	83	83	17	17	-
	35–43	82	85	15	15	-
	44–53	82	82	18	18	-
	≥ 54	84	89	10	11	-
Home language	-	-	-	-	-	0.26
	IsiXhosa	31	78	9	22	-
	IsiZulu	83	79	22	21	-
	Sesotho	43	84	8	16	-
	Setswana	32	89	4	11	-
	Tshivenda	37	93	3	7	-
	Tsonga	59	87	9	13	-
	Other	45	90	5	10	-
Highest level of education	-	-	-	-	-	0.02
	Primary	83	92	7	8	-
	Secondary	219	84	43	16	-
	Tertiary	28	74	10	26	-
Health facility	-	-	-	-	-	0.53
	Site 1	90	87	13	13	-
	Site 2	241	84	47	16	-
Employment	-	-	-	-	-	0.90
	Employed	143	86	24	14	-
	Unemployed	115	85	21	15	-
	Grant	7	88	1	12	-
	Pension	26	79	7	21	-
	Self-employed	38	84	7	16	-
Marital status	-	-	-	-	-	0.41
	Single	184	85	33	15	-
	Married	76	83	16	17	-
	Divorced	13	100	0	0	-
	Widowed	21	78	6	22	-
	Separated	36	88	5	12	-

Age, gender, language, health facility, employment and marital status did not show any significant association with SDM. Education was found to be a strong and significant predictor of SDM. Respondents with secondary education were nearly three times more likely to report SDM compared to those with only primary education (adjusted odds ratio [AOR] = 2.82, 95% CI: 1.10–7.23; *p* = 0.031). Those with tertiary education had the highest likelihood of SDM, with more than six times the odds compared to primary education (AOR = 6.28, 95% CI: 1.83–21.61; *p* = 0.004). Table 4 shows the univariate and multivariate analyses of factors influencing SDM.

**TABLE 4:** Multivariate logistic regression of factors influencing shared decision-making experience.

Characteristics	Level	Crude odds ratio	95% CI	*p*-value	Adjusted odds ratio	95% CI	*p*-value
Gender	Male	1.02	0.6–1.8	0.95	1.36	0.74–2.47	0.32
	Female (Ref)	-	-	-	-	-	-
Age (years)	≤ 34(Ref)	-	-	-	-	-	-
	35–43	0.89	0.42–1.90	0.77	0.91	0.40–2.06	0.82
	43–54	1.07	0.52–2.22	0.85	0.90	0.38–2.17	0.83
	≥ 54	0.60	0.26–1.38	0.22	0.64	0.20–1.98	0.44
Home language	IsiXhosa (Ref)	-	-	-	-	-	-
	IsiZulu	0.90	0.37–2.17	0.82	0.90	0.36–2.2	0.84
	Sesotho	0.64	0.22–1.85	0.41	0.69	0.23–2.03	0.50
	Setswana	0.43	0.12–1.54	0.20	0.42	0.11–1.55	0.19
	Tshivenda	0.28	0.07–1.12	0.07	0.27	0.07–1.15	0.08
	Tsonga	0.53	0.19–1.46	0.22	0.54	0.19–1.56	0.26
	Other	0.38	0.18–1.25	0.11	0.38	0.11–1.29	0.12
Highest level of education	Primary (Ref)	-	-	-	-	-	-
	Secondary	2.33	1–5.38	0.048	2.82	1.10–7.23	0.031
	Tertiary	4.23	1.47–12.1	0.007	6.28	1.83–21.61	0.004
Health facility	Site 1 (Ref)	-	-	-	-	-	-
	Site 2	1.22	0.64–2.34	0.53	1.49	0.75–2.98	0.26
Employment	Employed (Ref)	-	-	-	-	-	-
	Unemployed	1.00	0.56–1.78	0.90	1.07	0.58–1.99	0.82
Marital status	Single	0.9	0.4–1.6	0.60	0.69	0.30–1.57	0.38
	Married (Ref)	-	-	-	-	-	-
	Other	0.75	0.32–1.7	0.49	0.98	0.40–2.40	0.97

CI, confidence interval.

The most important facilitator reported by participants was ‘my doctor cares about me’ (54%). Other facilitators which participants thought had an influence on SDM were ‘My doctor made it clear that my input in the final treatment plan was important’ (37%) and ‘My doctor talks to me about my health and illness and explains things’ (30%). This does not imply that shared SDM occurred but that participants would be more inclined to participate in an SDM consult if the HCW allowed for open engagement and listened to suggestions made by the participant (Table 5).

**TABLE 5:** Highest-ranking barriers and facilitators.

Barriers and facilitators	Categories	Highest ranking	Frequency (*n*)	%
Barriers	Barrier 1	Long lines	95	24
	Barrier 2	Long waiting times	80	20
	Barrier 3	Poor staff attitude	99	25
Facilitators	Facilitator 1	My doctor cares about me	211	54
	Facilitator 2	My doctor talks to me about my health and illness and explains things	120	30
	Facilitator 3	My doctor made it clear that my input in the final treatment plan was important	144	37

The univariate logistic regression analysis found that none of the reported barriers had a statistically significant association with experiencing SDM. Patients who experienced long lines (*p* = 0.64), long waiting times (*p* = 0.18) or poor staff attitude (*p* = 0.83) were not significantly less likely to engage in SDM. Patients who felt that ‘my doctor cares about me’ (*p* = 0.63) or that ‘my doctor talks to me about my health and explains things’ (*p* = 0.205) did not show a statistically significant difference in SDM rates. However, the facilitator who said ‘my doctor made it clear that my input in the final treatment plan was important’ showed a strong association with SDM (*p* = 0.008). Long lines, long waiting times and poor staff attitude showed no significant association with SDM in AOR models. Perceiving that ‘my doctor cares about me’ (AOR = 0.86, 95% CI: 0.62–1.20; *p* = 0.38) and that ‘my doctor talks to me about my health and explains things’ (AOR = 0.78, 95% CI: 0.59–1.04; *p* = 0.086) were not significantly associated with SDM. However, patients who selected the most important facilitator of SDM in their experience as ‘my doctor made it clear that my input in the final treatment plan was important’ were significantly more likely to engage in SDM (AOR = 1.62, 95% CI: 0.42–0.92; *p* = 0.017), making it the only independent facilitator of SDM in this study.

## Discussion

This study aimed to determine the proportion of patients reporting SDM and the barriers, facilitators and sociodemographic determinants of patient engagement in SDM. The three main findings were the low number of patients who experienced SDM, the increased education level associated with a higher likelihood of engaging in SDM and the strong association between ‘My doctor made it clear that my input in the final treatment plan was important’ and SDM. Patients in the two Johannesburg clinics showed interest in wanting to engage with their HCWs during consultations, but SDM did not occur. There is documented evidence that SDM integration into undergraduate curricula is increasing.^41^ Despite this push towards SDM in undergraduate training, we found that only 32 patients (15%) reported SDM in their consultations.^19^ Previous studies done in South Africa revealed that if patients were given the opportunity, they would participate in SDM, but up to 40% of participants stated that they were never allowed to be involved in their treatment plan.^21,24^

Level of education showed a strong association with SDM in this study. A study conducted in Australia, which looked into health literacy and SDM, found that higher health literacy, often correlated with educational attainment, was linked to increased participation in SDM, which is in keeping with the findings of this study.^42^ Patients with more education were better able to understand medical information,^19^ ask questions, express preferences during consultations and challenge medical advice.^43^ According to the South African Human Rights Commission, access to information about patients’ rights has raised expectations for involvement in healthcare decisions, particularly among more educated individuals.^44^ Furthermore, patients with higher education levels reported more positive experiences with SDM, especially in outpatient settings where communication plays a central role.^45^ The South African healthcare system is challenging and riddled with barriers that can hamper SDM, such as low doctor–patient ratios, time constraints, under-resourced settings and doctors suffering burnout.^14,46^ While this study did not find significant associations between patient demographics, barriers or facilitators and SDM, other research identifies language, ethnicity, race, time constraints, and the power imbalance between patients and clinicians as obstacles to engaging in SDM.^46,47^ When doctors do not share the same socioeconomic status as their patients, they often struggle to relate to them. This power imbalance can hinder patients from communicating freely with their doctors.^48,49^ Healthcare workers have not been trained to be more culturally competent and to effectively manage the diversity of the South African background, thus hindering patient engagement by not being receptive to patients and their individual needs.^49,50^

This study found that if patients had a HCW who was willing to engage and involve them in the decision, then they were more likely to engage in the entire SDM process. This highlights that SDM does not only constitute deciding together but also involves analysing risks, benefits and outcomes of said decision. South Africa is guided by the Batho Pele Principles and the Patients’ Rights Charter,^51^ which advocates for patient-centred care. Encouraging active engagement in a consultation further promotes patient autonomy and improves understanding and adherence.^13,51^ This study emphasised the importance of the patient–HCW relationship and the HCW’s ability to create a positive experience during the consultation, allowing for active patient engagement and SDM. With the steady improvement of education levels in South Africa and access to information, SDM becomes a non-negotiable skill.

### Study limitations

A strength in this study was that recall bias was minimised. Questionnaires were in English and verbally translated for people who struggled to understand. The use of visual aids was helpful in this regard, as everyone had the same image to decide on; however, in the translation, meaning could be lost. The study only addressed SDM from a patient perspective and not the HCW perspective. Response bias and social desirability bias must be considered, as these patients do not have the option of other health care facilities. The study’s sample showed a similar distribution of patient demographics compared to probability and non-probability studies done in the South African PHC setting.^21,52^ A comparison of patient demographics in this study to demographics of the Chiawelo and Alexandra communities shows that age, gender, most widely used language and employment were similar.^53,54^ Selection bias was minimised by utilising consecutive sampling, sampling from more than one site, and consistent and validated data collection tools.^36^ Although these factors do not eliminate the selection bias associated with convenience sampling, they do minimise it. The barriers and facilitators tool only allowed patients to choose from a predetermined list of options. The reason why researchers chose to include predetermined options was to avoid coding inconsistencies and difficulties; however, including open-ended questions in this section would have increased the study’s depth.^55^

### Recommendations

Qualitative studies in the future may render good insights into patient and HCW perspectives on SDM. Leaders in PHC and stakeholders must focus on system issues that prevent patients from engaging in SDM. Undergraduate and postgraduate training in family medicine should not only advocate for SDM but also focus on how to implement SDM in busy work environments. Patient education via social media, radio stations and newspapers regarding SDM should be advocated for.

## Conclusion

A great majority of patients reported that SDM did not occur during consultations with their healthcare providers (85%). A total of 37% of patients wanted to be involved in SDM, but this research suggests that sociodemographic factors, such as level of education, and system challenges, long waiting times (20%) and poor staff attitude (25%), influenced patients’ experiences of SDM. The association between the facilitator’s statement ‘My doctor made it clear that my input in the final treatment plan was important’ and SDM was similar to the findings of Levinson et al., which found that 96% of patients wanted to be given information and asked their opinion on their treatment plan.^19^ This study can be utilised to strengthen undergraduate and postgraduate training around SDM. In the PHC setting, it can be used to strengthen system issues and implement quality improvement projects which focus on addressing the major barriers to SDM. It can also be used to further explore SDM from an HCW perspective.
